# Neuroprotection Caused by Hyperoxia Preconditioning in Animal Stroke Models

**DOI:** 10.1100/tsw.2011.23

**Published:** 2011-02-14

**Authors:** Mohammad Reza Bigdeli

**Affiliations:** Faculty of Biological Sciences, Shahid Beheshti University, G.C. Tehran, Iran

## Abstract

Ischemic tolerance induced by hyperoxia (HO) can protect against brain injury and neurodegenerative diseases. Although multiple studies demonstrate neuroprotection by HO, the exact mechanism(s) of HO neuroprotection has not been elucidated. Here, I first review related mechanisms of brain ischemia and then data evaluating the neuroprotective effects of HO in focal and global ischemic animal models. I clearly establish that the cerebrovascular, extracellular matrix, plasma membrane, endoplasmic reticulum, mitochondrial, and lysosomal reactions are critical in neuroprotection induced by HO in focal ischemia. In rats and mice, the middle cerebral artery occlusion (MCAO) model was used to represent cerebrovascular stroke. Neuroprotection induced by HO exhibits specific adaptation responses that involve a number of cellular and biochemical alterations, including metabolic homeostasis and reprogramming of gene expression. The changes in the metabolic pathways are generally short lived and reversible, while the consequences of gene expression are a long-term process and may lead to the permanent alteration in the pattern of gene expression. The neuroprotection provided by HO may have important clinical implications. Therefore, it is important to assess the benefits and risks of HO therapy in noninfarcted tissue.

## INTRODUCTION

Ischemic-reperfusion injury is a major complication occurring in brain stroke, aneurysm surgery, sickle cell anemia, congenital heart defects, heart attack, and neurotransplantation. There are two components in brain ischemia: hypoxia and vascular responses. One of the vascular responses of central nervous system (CNS) damage after cerebral ischemia is the formation of brain edema caused by the breakdown of the blood brain barrier (BBB)[1]. Hypoxia interrupts the energy metabolism of the brain, which in turn results in increased endothelial cell permeability and edema function, and then leads to neurological deficits[2]. The endothelin-1 level is increased in the cerebral spinal fluid (CSF) and plasma in stroke patients, and in a stroke animal model[3]. Superoxide dismutase prevents vasogenic brain edema after several kinds of injuries[4,5], suggesting that the superoxide anion is an important factor for disruption of barrier function (Fig. 1). Superoxide dismutase is a metalloenzyme that catalyzes the dismutation of the superoxide anion into $O_2$ and hydrogen peroxide ($H_2O_2$) in the cytosol, mitochondria, and nucleus[6,7]. Subsequently, $H_2O_2$ is reduced to $H_2O$ by glutathione peroxidase in the cytosol, or by catalase in the peroxisomes or in the

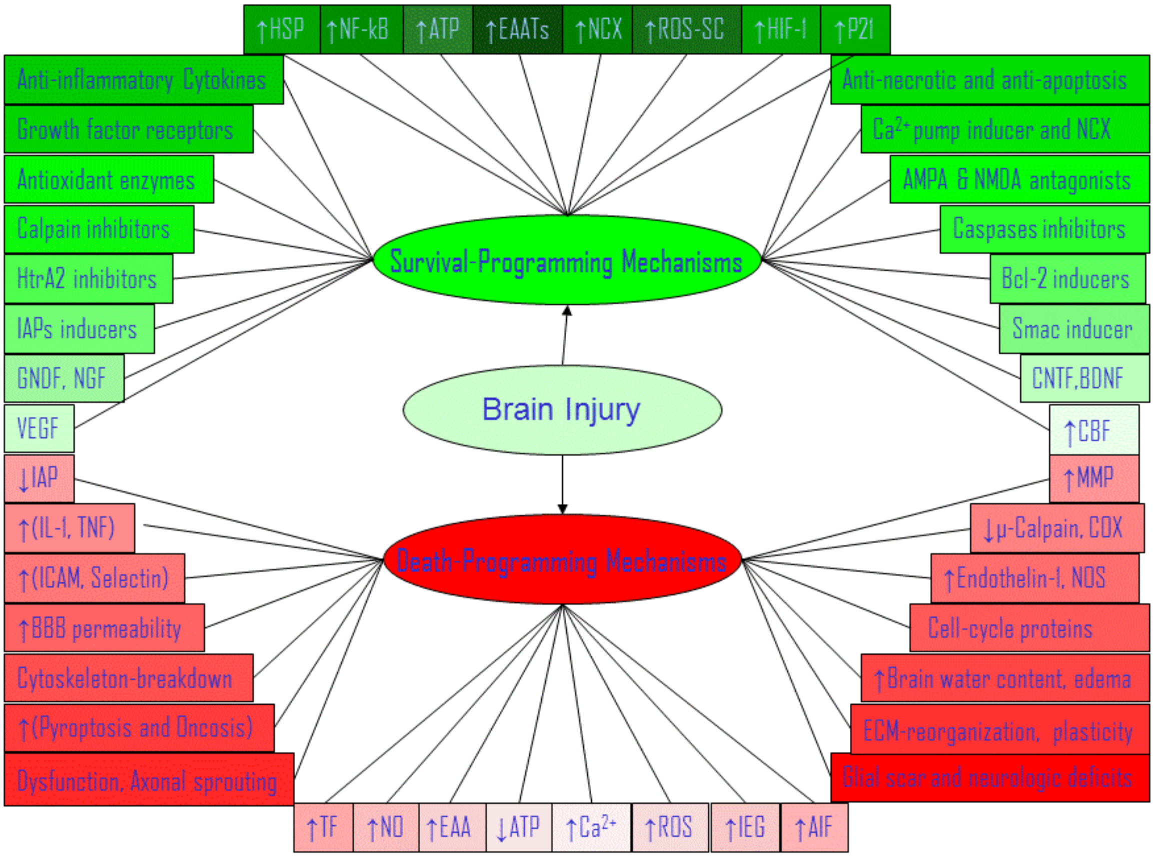

**FIGURE 1.** Temporal (color intensity) and schematic showing target events by which ischemia proteomics (brain injury) could either protect or enhance cell death dose dependently. Abbreviations: AIF, apoptosis-inducing factor; AMPA, α-amino-3-hydroxyl-5-methyl-4-isoxazole-propionate; ATP, adenosine triphosphate; BBB, blood brain barrier; BDNF, brain-derived neurotrophic factor; CBF, cerebral blood flow; IAPs, the inhibitor of apoptosis proteins, XIAP, cIAP-1, cIAP-2, and survivin; CNTF, ciliary neurotrophic factor; COX, cyclo-oxygenase; EAA, excitatory amino acid; EAAT, excitatory amino acid transporter; ECM, extracellular matrix; GDNF, glial cell line–derived neurotrophic factor; HIF-1, hypoxia-inducible factor-1; HSP, heat shock protein; HtrA2, the mature serine protease Omi; ICAM, intracellular adhesion molecule-2; IEG, immediate early gene; IL-1, interleukin-1; MMP, matrix metalloproteinase; NCX, Na-Ca exchanger; NF-κB, nuclear factor-kappa B; NGF, nerve growth factor; NMDA, N-methyl-D-aspartic acid; NO, nitric oxide; NOS, nitric oxide synthase; P21, potent cyclin-dependent kinase inhibitor CKI; ROS-SC, ROS scavenger in the cell; Smac, the mitochondrial protein that binds to IAPs and deactivates them; TF, tissue factor; TNF, tumor necrosis factor; VEGF, vascular endothelial growth factor.

cytosol. Another manifestation of CNS damage is the direct injury of the neural cell, including excitatory events that are induced by glutamate release after cerebral ischemia[8]. Glutamate elevates free calcium ($Ca^{2+}$), which activates $Ca^{2+}$-dependent enzymes and leads to free radical production[9]. Therefore, if calcium levels, either extracellular or intracellular, become too high or too low, disruption of the BBB will occur. In this way, regulation of calcium homeostasis seems to be critical[10].

A noxious stimulus applied at doses close to, but below the threshold of, cell injury induces an adaptive response that protects the brain against additional stress from the same (tolerance) or other (cross-tolerance) stimuli. Ischemic tolerance (IT) is an endogenous phenomenon that can result in subsequent resistance to severe ischemic injury. This phenomenon has been reported in several organs, including the brain and kidneys[11,12,13]. Among different stresses, hypoxia[14], ischemia[15], anoxia[16], oxidative stress[17], and inhibitors of oxidative phosphorylation[18] induce tolerance to subsequent cerebral (focal or global) ischemia.

Most of such stimuli, however, lack the potential for clinical translation due to associated toxicity. For this reason, safe nonpharmacological stimuli have been studied extensively. Recently, similar protection has been shown to be conferred by hyperoxia (HO)[19] and hyperbaric oxygen (HBO)[20], perhaps (as in other situations[21]) through the generation of oxygen free radicals and hydroxyl radicals[22].

Several possible, underlying, related mechanisms of induction and maintenance of IT in the brain have been suggested: NMDA receptors[23], antiapoptotic factors[24], IL-1[25], superoxide dismutase[15], reactive oxygen species (ROS)[21], nitric oxide–dependent p21-ras activation[26], metallothioneins[27], activation of VEGF receptor and Akt[28], erythropoietin[29], caspase-3[30], and NF-κB and proinflammatory cytokines[31]. Recent studies suggest that normobaric HO results in IT to reduce ischemic brain injury[32,33]. The neuroprotection results of HO may have important clinical implications (Fig. 1). Therefore, it is important to assess the benefits and risks of HO therapy in noninfarcted tissue[32]. For example, it has been shown that the continued exposure to ROS generated by oxidative metabolism or environmental sources can exert critical cellular structure damages and be responsible for the cell death process[34].

During physiological conditions, ion transporters on the microvessel endothelial cells control ion flux across the BBB. The transport of other molecules occurs via specific transporters or transcytosis, while paracellular diffusion is tightly regulated. In pathological conditions, including stroke[35], diabetes[36], Alzheimer's disease[36], multiple sclerosis[37], and inflammatory pain[38], there is a disturbance of BBB integrity, with disruption of ion homeostasis and transporter function. In stroke, this loss of ion regulation is followed by the passive diffusion of water, resulting in brain edema.

Recent investigations have demonstrated that HO preconditioning triggers a signaling pathway by potentiating TNF-α. The signal transduction involves NF-κB, which subsequently transmits the signal via the activation of related gene expression. Our results clearly indicated a role for TNF-α, because other investigations have shown that inhibition of the TNF-α receptor almost partly blocked the neuroprotection. Subsequently, the role of antioxidant enzymes as one of the potential targets for induction of IT has been shown (Fig. 1). Recently, oxygen-derived free radicals have been implicated in the transmembrane signaling process[39].

This review will focus on the molecular mechanisms of HO preconditioning. The results from our laboratory and other laboratories suggest that HO and ROS function as second messengers during induction of IT.

## ACTIVATED PROTEIN C (APC) AND STROKE

PC is a vitamin K–dependent plasma glycoprotein that is synthesized by the liver and circulates as a 2-chain biologically inactive species. It is transformed to its active form, APC, by thrombin-mediated cleavage of PC at the N-terminal. Effective activation of PC by thrombin requires the transmembrane glycoprotein thrombomodulin (TM) as a cofactor for thrombin[40]. Breakdown of the BBB plays a key role in inflammatory disorders such as stroke. The blood coagulation protease thrombin has proinflammatory effects on endothelial cells and is a well-characterized barrier disruptive factor[41]. In contrast, the bioactive lipid sphingosine 1-phosphate (S1P) recently has been demonstrated to have potent barrier-protective effects in low levels of APC, but not in high levels[42]. The opposite effects of thrombin and S1P on endothelial barrier integrity are both mediated by G protein–coupled receptors, i.e., protease-activated receptors (PARs)[43] and S1P receptors[44], respectively (Fig. 2-A1). Current information indicates that protein-activated receptor-1 (PAR1) is the main receptor responsible for thrombin signaling in endothelial cells, whereas endothelial cell PAR2 is not activated by thrombin, but by several other proteases, including trypsin and mast cell tryptase[45]. BBB protection by APC proceeds via unanticipated cross-talk between the prototypical barrier-disruptive PAR1 and barrier-protective S1P

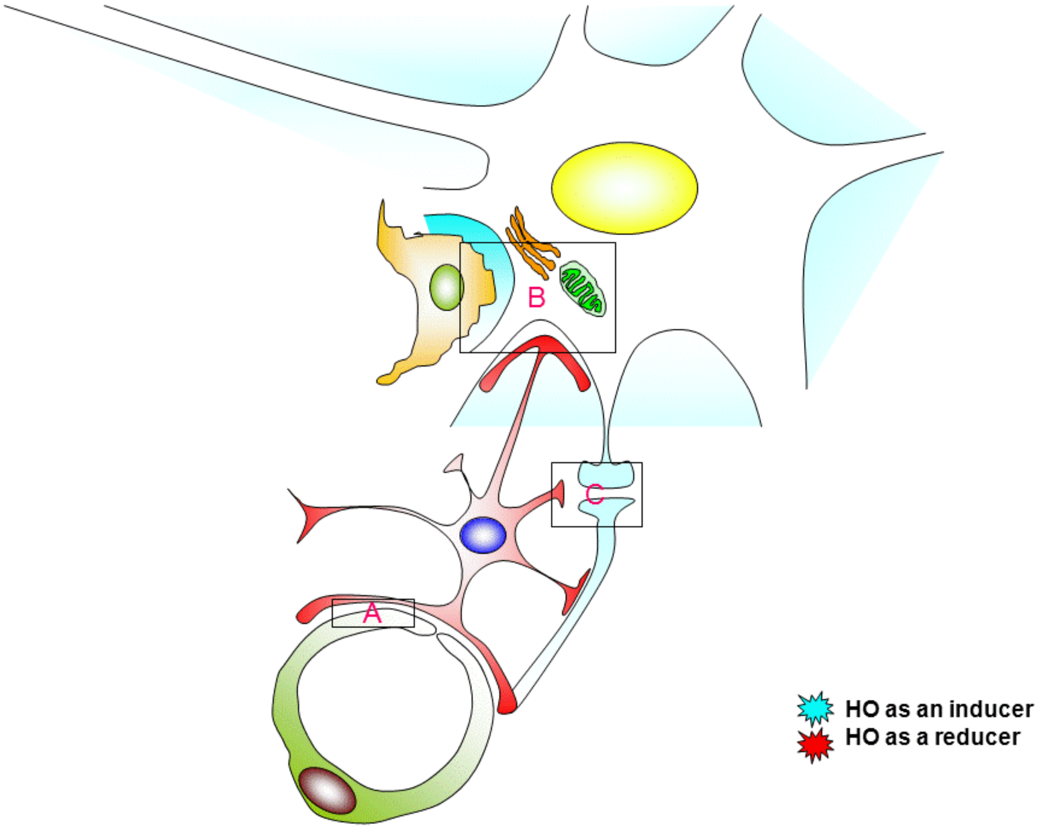

**FIGURE 2.** Extra- and intracellular signaling in postischemic brain injuries. See also Figs. 2-A1, 2-A2, 2-B, and 2-C.

signaling pathway. Therefore, it has been demonstrated that cell survival is induced by inhibition of P53 production. PAR1 activation by higher concentrations of thrombin leads to barrier disruption, whereas barrier protection by APC or low concentrations of thrombin are mediated by sphingosine kinase-1 activity and cross-activation of S1P1 signaling (Fig. 2-A1). In addition, PAR1 and the endothelial protein C receptor (EPCR) are essential for the ability of APC to protect the brain endothelium from ischemic injury. However, cleavage and activation of PARs by extracellular proteases are capable of causing intracellular $Ca^{2+}$ signaling[46]. Although APC is neuroprotective and appears to protect stressed brain endothelial cells from ischemic injury, involvement of neuroprotection induced by HO and the role of APC under physiological conditions remain to be elucidated.

## ENDOTHELIN AND STROKE

Endothelin (ET)-1, a 21-amino-acid peptide originally isolated from porcine aortic endothelial cells, is one of the most potent vasoconstrictors known[47]. It exerts various physiological actions by binding to two specific G protein–coupled receptor subtypes, $ET_A$ and $ET_B$ receptors, in mammalian cells[48]. The ET-1 level is increased in the CSF and plasma in stroke patients[3]. Cerebral ischemia is mainly caused by the occlusion of the cerebral artery, either by embolus or by local thrombosis. Several humoral factors caused by ischemia, including ET-1, have been shown to increase BBB permeability[49]. Recently, increased synthesis of ET-1 was observed in endothelial cells and astrocytes under ischemic conditions[50]. Alternatively, abluminal application of ET-1 to the middle cerebral artery was used as an ischemic model to reduce blood flow[51].

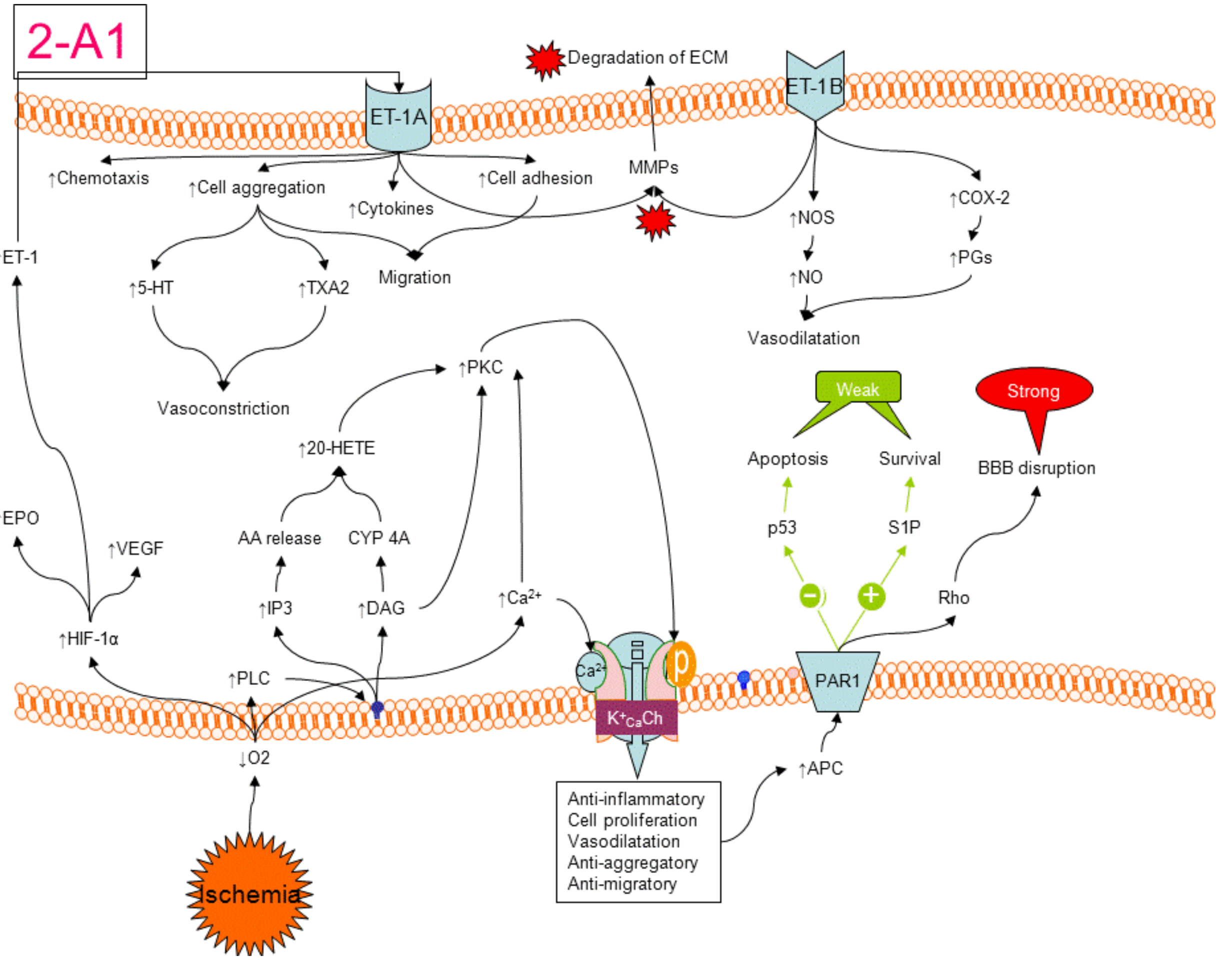

**FIGURE 2-A1.** Injuries caused by ischemia occur in a wide range of subcellular regions, including the capillary endothelium in nongenomic levels (green arrow: neuroprotective; red burst: neurodegenerative). Abbreviations not listed for previous figures: 5-HT, 5-hydroxytryptamine; AA, arachidonic acid; APC, activated protein C; CYP; cyclophilin; DAG, diacyl glycerol; EPO, erythropoietin; ET-1, endothelin-1 A and B; HETE, hydroxyeicosatetraenoic acid (lipoxygenase products); IP3, inositol triphosphate; $K^{+}_{Ca}Ch$, $Ca^{2+}$-dependent K channel; PAR1, protein-activated receptor 1; PG, prostaglandin; PKC, protein kinase C; PLC, phospholipase C; S1P, sphingosine 1-phosphate; TXA2, thromboxane A2.

A number of factors contribute to vascular damage after ischemic-reperfusion injury, including accumulation of oxygen free radicals[52], nitric oxide (NO), and matrix metalloproteinase-2 (MMP-2), at early stages of stroke[49,53,54]. ET-1 is a potent activator of superoxide production in vascular smooth muscle cells through $ET_A$ and $ET_B$ receptors via human umbilical vein endothelial cells (Fig. 2-A1).

The effect of ET-1 on apoptosis is controversial. ET-1 has been reported to be an antiapoptotic factor in endothelial cells[55]. On the other hand, there are some studies about smooth muscle cells in which ET-1 causes apoptosis[48]. $ET_A$ antagonists significantly reduced intercellular adhesion molecule-1 (ICAM-1) and PAR1 immunoreactive vessels in ischemic boundary regions, when they were administered 2 h after embolic stroke. Stroke induces adhesion molecule expression, including ICAM-1, in cerebral vessels, which facilitates leukocyte adhesion. When neutrophils adhere to the injured endothelium, they interact with platelets and form clots that contribute to secondary thrombosis in cerebral microvessels and disruption of the BBB[56]. Moreover, Zhang et al. suggest that $ET_A$ may be involved in PAR1 expression

(Fig. 2-A1). PAR1 is the prototype thrombin receptor, which is activated by APC, which also promotes thrombosis formation[57]. The distribution of ET-1 and its receptors $ET_A$ and $ET_B$ in different sites of the CNS suggests that it may influence a wide range of CNS-controlled functions. Under pathological conditions of CNS, such as stroke, ischemia-reperfusion and infarction, subarachnoid hemorrhage, and Alzheimer's disease, ET-like immunoreactivity has been detected in astrocytes of the brain (Fig. 2-A2)[58]. Although $ET_A$ antagonists are neuroprotective and appear to protect stressed brain endothelial cells from ischemic injury, involvement of neuroprotection-induced HO and the synergism effect of the $ET_A$ antagonist under physiological conditions remain to be elucidated.

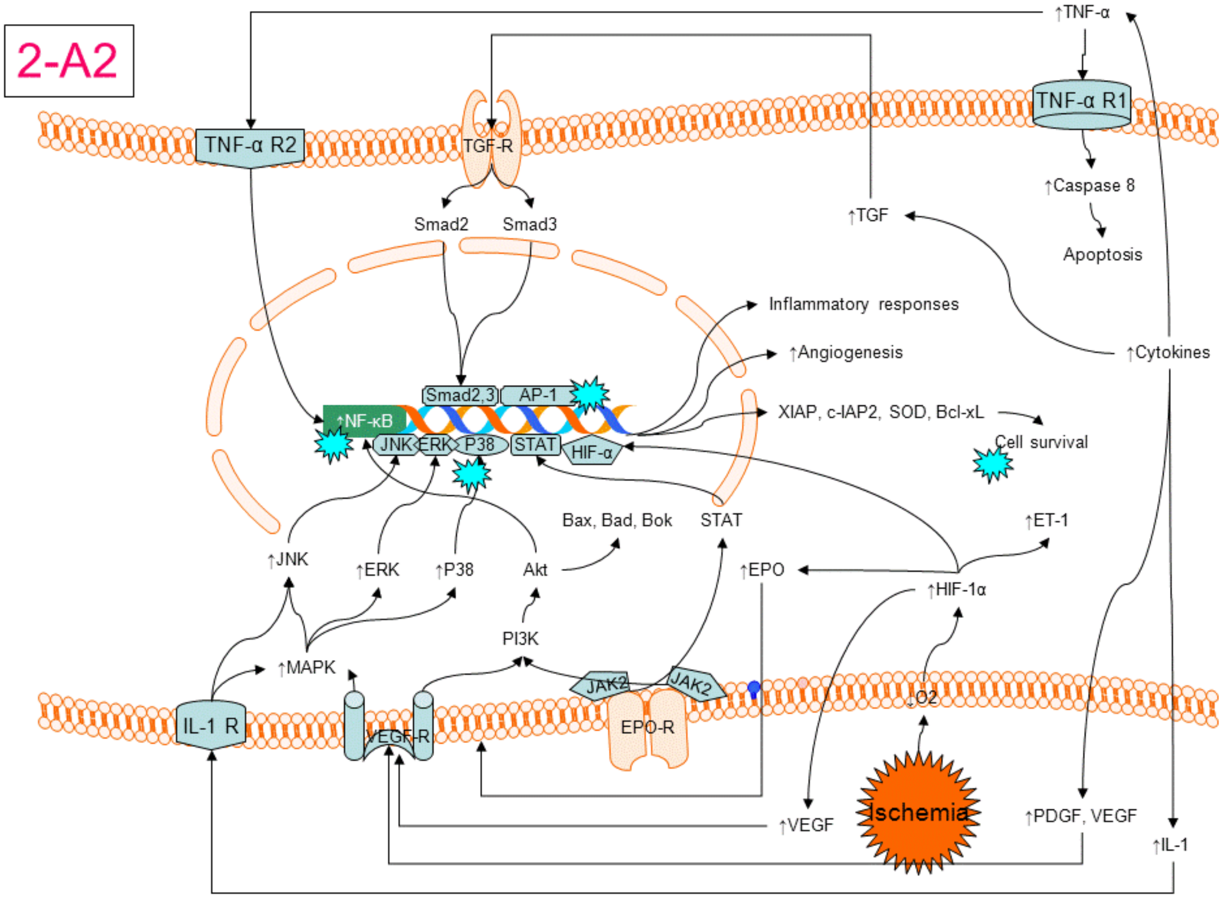

**FIGURE 2-A2.** Ischemic injuries occur in the capillary endothelium in genomic levels (green burst: neuroprotective). Abbreviations not listed for previous figures: Akt, protein kinase B; AP-1, activated protein; Bad, Bcl-xL/Bcl-2-associated death promoter; Bax, Bcl2-associated X protein; Bok, Bcl2-related ovarian killer; EPO-R, erythropoietin receptor; ERK, extracellular signal-regulated kinase; IL-1-R, interleukin-1 R; JAK, just another kinase (Janus kinase); JNK, Jun N-terminal kinase; MAPK, mitogen-activated protein kinase; PDGF, platelet-derived growth factor; PI3K, phosphoinositide 3-kinase; Smad, Smad-dependant apoptosis induced by transforming growth factor-β (TGF-β); SOD, superoxide dismutase; STAT, signal transducer and activator transcription; TGF-R, TGF receptor; VEGF-R, VEGF receptor.

## CELL DEATH AND STROKE

Cerebral ischemia provides conditions in which there is insufficient brain microcirculation to meet metabolic demands. Brain ischemia leads to cerebral infarction and stroke. Rapid reduction of ATP

results in the absence of biochemical energy and the reduction of electrochemical gradients. Therefore, there is a massive calcium influx into the cytosol that leads to massive release of glutamate from synaptic vesicles, lipolysis, proteolysis, calpain activation, reduction of protein synthesis, and accumulation of metabolic wastes[59].

Prolonged hypoxia induces cell death via apoptosis and necrosis. Apoptotic changes (cytoskeleton breakdown and absence of cell membrane asymmetry) recall microglia and macrophages, cell shrinkage, and DNA fragmentation (Fig. 2-A2). In contrast to apoptosis, necrosis results from acute cellular injury. Damage to DNA from ROS, oxidative stress, and ischemia can induce apoptosis via the action of the tumor-suppressing gene P53[60].

The mechanism of apoptosis is regulated by various cell signals, which may initiate either extrinsic or intrinsic pathways. Growth factors, NO, and cytokines, which are produced by cerebral ischemia, can induce the extrinsic pathway through either cross-membrane or transduction to effect a response (Fig. 1). Cerebral ischemia, ROS, hypoxia, and increased intracellular $Ca^{2+}$ levels can trigger the intrinsic pathway. On the other hand, poly-ADP ribose polymerase (PARP) may regulate apoptosis (Fig. 1). Apoptotic proteins can cause mitochondrial swelling via the formation of the mitochondrial permeability transition pore (PTP; Fig. 2-B). Extrinsic and intrinsic pathways lead to apoptosis through dissipation of mitochondrial membrane potential and then make it more permeable[61].

The second mitochondrial-derived activator of caspases, Smac, is released into the cytosol via PTP. Smac binds to the inhibitor of apoptotic proteins (IAPs) and deactivates them. They normally suppress the activities of caspases (Fig. 1). Cytochrome C is also released from PTP (Fig. 2-B). It binds to Apaf-1, which then binds to procaspase-9 and activates caspase-3. Bcl2 proteins can inhibit apoptosis either by direct action on PTP or indirectly via other proteins (Fig. 2-B).

Direct apoptotic signal transduction is mediated through two pathways: a TNF-induced model and a Fas-Fas ligand–mediated model, both involving receptors of TNF (TNF-R). Most cells have two receptors for TNF-α: TNF-R1 and TNF-R2 (Fig. 2-B, A2). Binding of TNF-α to TNF-R has been shown to initiate both caspase activation and survival, and inflammatory responses[62]. Although the mechanism of neuroprotection induced by HO has not been clarified, HO can induce gene expression of antiapoptotic proteins, including Bcl2, HIF, TNF-α, and antioxidant enzymes, partly by triggering the TACE/TNF-α/NF-κB pathways (Fig. 2-A2).

## ROS AND HO

Prolonged exposure to HO leads to the generation of excessive ROS. On the other hand, there is some evidence showing that ROS act as signaling molecules, including involvement of ROS and Ca signaling. Recent studies show that the infarct volume was abolished by DMTU, a hydroxyl radical scavenger, suggesting that ROS play crucial roles in preconditioning[39]. Neural tissue is susceptible to oxidative damage because of the brain's high oxygen consumption and concentration of oxidizable substances, such as catecholamines and ascorbic acid or lipids[63]. Oxidative stress resulting from the generation of ROS and NO is present in the brain after ischemia, hypoxia, and HO[5].

It has been shown that treatment with the mito-$K_{ATP}$ channel opener diazoxide is able to act as a trigger of preconditioning in the cardiomyocytes[64]. Alternatively, evidence has demonstrated that mito-$K_{ATP}$ channels reside upstream of kinases in the signal transduction pathway. Finally, it was found that diazoxide confers its protection through free radicals, a known trigger for preconditioning[65]. Pain et al. proposed that the opening of these channels during ischemic preconditioning generates free radicals, which then triggers the IPC memory. Then, during the subsequent ischemic insult, a kinase cascade modulates some unidentified end effector that actually protects the heart.

Several studies have demonstrated that free radical signaling plays a central role in ischemic preconditioning. Therefore, brain ischemic preconditioning was blocked by DMTU and inhibited preconditioning-mediated phosphorylation of p38 mitogen-activated protein kinase (MAPK) and MAPKAP kinase 2 activity ([39]; Fig. 2-A2). Cardioprotection-mediated nuclear translocation and activation

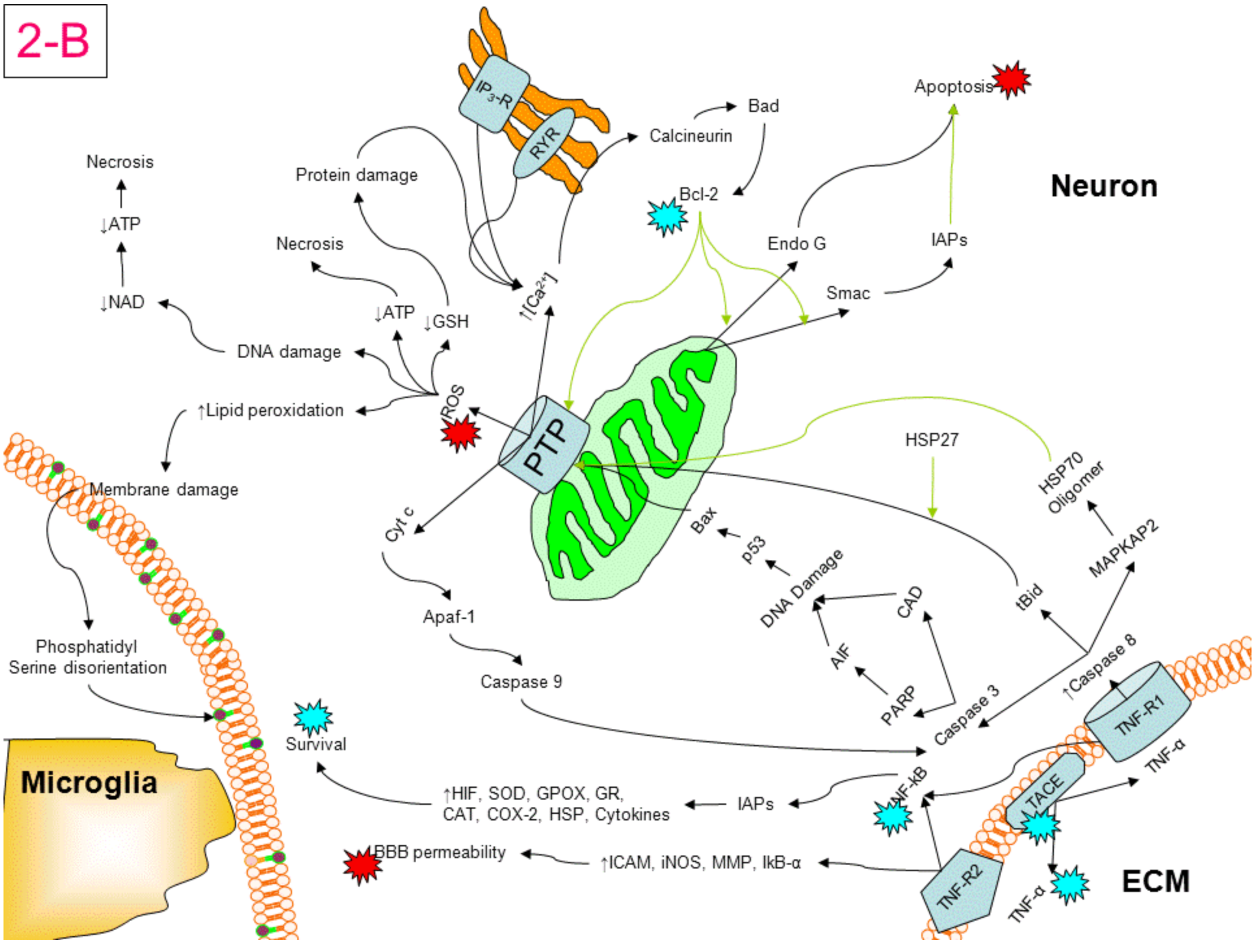

**FIGURE 2-B.** Injuries caused by ischemia occur in a wide range of subcellular regions of the neuron (plasma membrane, endoplasmic reticulum, and mitochondria; green arrow: neuroprotective, red burst: neurodegenerative). Abbreviations not listed for previous figures: Apaf, antiapoptosis protease activating factor; CAD, caspase-activated DNase; CAT, catalase; Cyt c, cytochrome c; Endo G, endonuclease G; GPOX, glutathione peroxidase; GR, glutathione reductase; GSH, glutathione; IkB-α, inhibitor of NF-κB; iNOS, inducible nitric-oxide synthase; MAPKAP2, phosphorylation of MAPK-activated protein kinase 2; NAD, nicotinamide adenine dinucleotide; PARP, poly (ADP-ribose) polymerase; PTP, permeability transition pore; RYR, ryanodine receptor; TACE, TNF-α converting enzyme; tBid, truncated of Bid; TNF-R1 and R2, TGF-a receptor1 and 2.

of NF-κB was blocked by DMTU[39]. Therefore, current results indicate that ROS provoke a signal transduction pathway through the activation of G protein and tyrosine kinase phosphorylation. Then, their activities are promoted independent of PKC or act downstream of PKC. It has been shown that the effects of HBO in reducing infarct volume were not correlated with the restoration of local cerebral blood flow during ischemia and were reversed by administration of 5-hydroxydecanovate or glibenclamide[66]. On the other hand, it has been shown that HO improved mitochondrial functions and loss of $\Delta\psi_M$ is largely restored by HBO[67]. Therefore, HO and HBO have been shown to promote neuronal survival, reduce oxidative stress[22], modulate neuroinflammation[68], and decrease apoptosis-related death[69,70].

## HO AND EAATS

Intermittent and prolonged HO preconditioning has been shown to confer neuroprotection against ischemic-reperfusion injury[1]. IT also decreases excitotoxicity via reduction of extracellular glutamate in the rat brain[71,72]. Recently, it has been suggested that preconditioning with prolonged and intermittent normobaric HO up-regulates glutamate transporters (EAAT1, EAAT2, and EAAT3) in the rat brain and increases the serum TNF-α level. These results show that IT caused by intermittent and prolonged HO can occur, at least partly, due to EAAT expression. Glutamate is known to play a predominant role in the pathogenesis of ischemic brain injury. This excitatory amino acid is released in high concentrations in the core of the cerebral infarction and in the penumbral tissue, where it overactivates glutamate receptors (NMDA and AMPA), leading to influx of $Ca^{2+}$ and $Na^{+}$ that activate a variety of processes, such as protease activation, lipase activation, increase of free radicals, altered transcription, PKC activation, hydrolysis of protein phosphatase inhibitor (PPI), and cellular swelling that subsequently produces cell death[73](Fig. 2-C). Therefore, glutamate transporters and Na-Ca exchangers are potential targets for ischemic preconditioning[74].

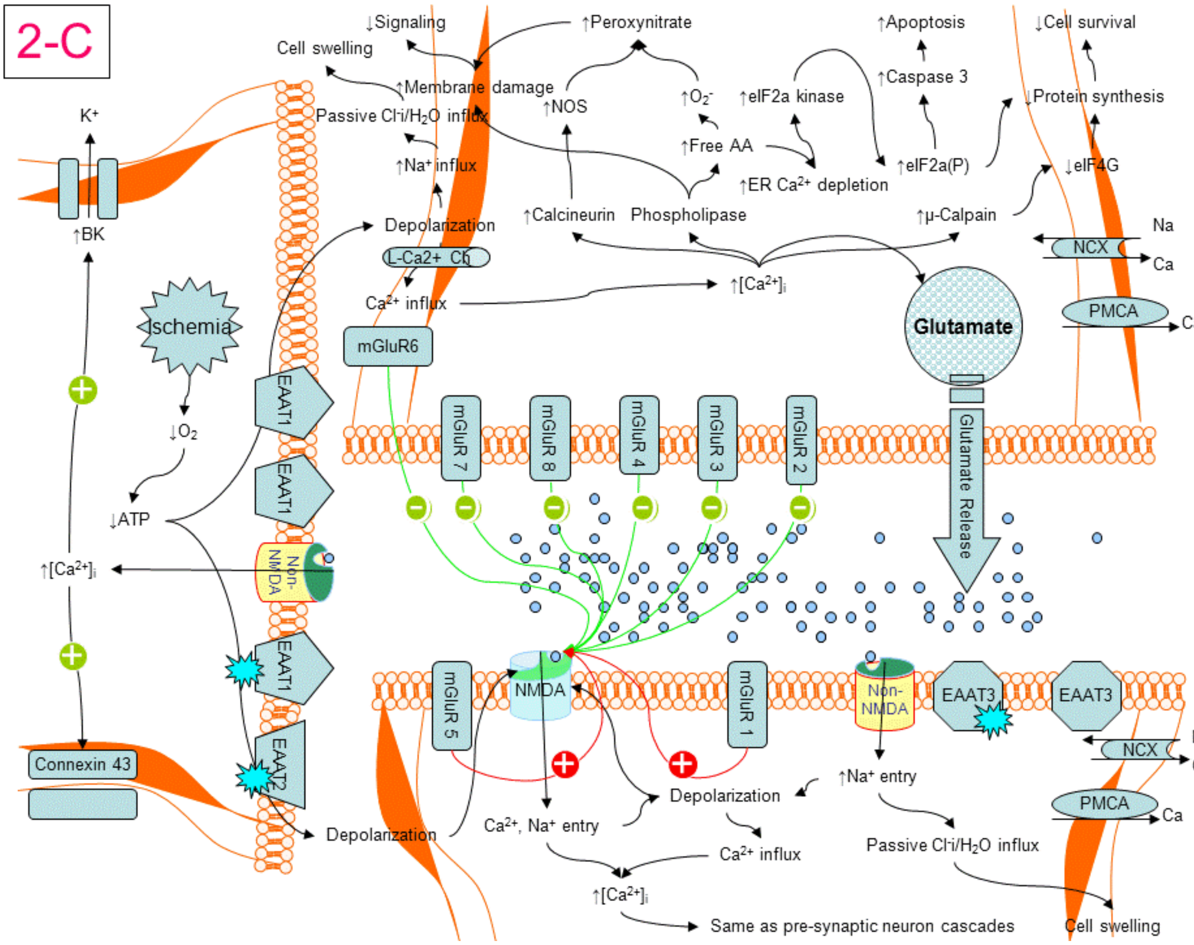

**FIGURE 2-C.** Injuries caused by ischemia occur in a wide range of subcellular regions of the synapse and astrocyte (green arrow: neuroprotective, red arrow: neurodegenerative). Abbreviations not listed for previous figures: BK, background K channel; eIF2a (P), phosphorylated eukaryotic initiation factor 2-a; eIF4G, eukaryotic initiation factor 4-G; ER, endoplasmic reticulum; L-$Ca^{2+}$ Ch, L-type $Ca^{2+}$ channel; mGluR, metabotropic glutamate receptor; PMCA, plasma membrane $Ca^{2+}$ ATPase.

Glutamate transporters in neurons and glia remove glutamate from the extracellular space, thereby helping to terminate glutaminergic synaptic transmission and to prevent the extracellular glutamate concentration from rising to neurotoxic levels[75,76]. Up to now, six high-affinity, sodium-dependent glutamate transporters have been cloned[77]. EAAT1/GLAST and EAAT2/GLT1 are localized primarily in astrocytes. EAAT3/EAAC1 and EAAT4 are distributed in neuronal membranes (Fig. 2-B). EAAT5 is located in the retina and ecgltp in *Escherichia coli*.

Intermittent and prolonged HO has also been shown to increase the expression of EAAT/GLAST in the nonischemic hemisphere. Cellular expression of EAAT1/GLAST is induced by HO, whereas it is not increased by ischemia[73]. In this way, preconditioning with intermittent and prolonged HO may induce expression of glutamate transporters (especially EAAT1) by different molecular pathways. Yamashita et al. examined the *in vivo* role of EAAT4 and EAAT1/GLAST in protecting Purkinje cells from ischemic-induced damage. Purkinje cells of EAAT1/GLAST mutant mice were more vulnerable to ischemia than those of EAAT4 mutant mice[78]. These results demonstrate that EAAT1/GLAST plays a role in preventing excitotoxic cerebellar damage after ischemia in concert with EAAT4[78]. Therefore, although all glutamate transporters are potentially involved in the protection of neurons following ischemia, EAAT1/GLAST appears to play a dominant role during ischemia (Fig. 2-C).

Intermittent and prolonged HO has also been revealed to increase the expression of EAAT3/EAAC1 in the nonischemic hemisphere[59]. It has also been demonstrated that EAAT2 is up-regulated after ischemic preconditioning and TNFR1 does not mediate increased EAAT2 expression after cerebral ischemic preconditioning[74]. EAAT2 has been reported to be the main glutamate transporter, since it is responsible for the bulk of glutamate clearance in the rat cerebral cortex[79]. Gene deletion of EAAT2 was associated with larger increases in extracellular glutamate, neural damage, and brain edema after experimental brain ischemia[80]. Therefore, the EAAT2 expression stimulator may be useful in preventing ischemic neural damage. Intermittent and prolonged HO has also been demonstrated to increase the expression of EAAT3/EAAC1 in the nonischemic hemisphere. Expression of EAAT3 is weak in intermittent HO, and alteration of EAAT3 expression parallels the serum TNF-α level (Fig. 2-A2; [19,59]). Recently, it has been shown that unlike GLAST and GLT1, neuronal EAAC1 does not play a major role in clearing glutamate from the extracellular space. Instead, EAAC1 can transport cysteine, an obligate precursor for glutathione synthesis, far more effectively than GLAST and GLT1[81]. It was also reported that EAAC1 deficiency leads to impaired neuronal glutathione metabolism and age-dependent brain atrophy. Therefore, increased expression of EAAT3 via the TACE/TNF-α pathway is useful to decrease extracellular glutamate concentration and provide an obligate precursor of glutathione synthesis by serum TNF-α levels.

Alternatively, there are metabotropic glutamate receptors (mGluR) that indirectly activate ion channels on the plasma membrane through G protein. The mGlutR can be divided into three main groups[82]. The mGlutR in group I, including mGlutR1 and mGlutR5, which are mainly postsynaptic, increase NMDA receptor activity and risk of excitotoxicity[83]. The mGlutR in group II, including mGlutR2 and mGlutR3, which are mainly presynaptic, decrease NMDA receptor activity and risk of excitotoxicity. The mGlutR in group III, including mGlutR4, mGlutR6, mGlutR7, and mGlutR8, which are mainly presynaptic, decrease NMDA receptor activity and risk of excitotoxicity[84](Fig. 2-C). Although mGlutR is important in excitotoxicity, involvement of their HO preconditioning under physiological conditions remains to be elucidated (Fig. 2-C).

## HO AND TNF

We previously showed that serum TNF-α levels were increased in intermittent and prolonged HO groups[59]. Alternatively, it has been demonstrated that TACE is up-regulated after ischemic brain damage. The increase in TACE expression contributes to the rise in TNF-α and subsequent neuroprotection effects after excitotoxic stimuli[85,86]. It is well known that TACE-induced TNF-α release participates in the development of IT, because incubation of ischemic preconditioning–exposed cells with TACE inhibitor or anti–TNF-α antibody is able to inhibit ischemic preconditioning–induced neuroprotection[73]. HO-induced neuroprotection may involve up-regulation of glutamate transporters partly mediated by the TACE/TNF-α pathway[19]. On the other hand, it has been shown that TNFR1 mediates increased neuronal membrane EAAT3 expression after cerebral ischemic preconditioning[74] (Fig. 2-B). The increased expression of EAAT3 after ischemia might be partly dependent on the TACE/TNF-α pathway[73,74].

## TNF AND NF-κB

Perturbation of the cellular redox equilibrium appears to be central for the activation of AP-1 and NF-κB. Therefore, AP-1 induction by oxidative stress can be reversed by free radical scavengers, suggesting a common intermediate and long signaling redox pathway (Fig. 2-A2)[87]. Choi et al.[88] have demonstrated enhanced NF-κB activity and Bcl2 expression through ROS and PKC-dependent signaling pathways in both ischemic and HO preconditioning. They also found that NF-κB inhibitors abrogated the infarct-limiting effect shown in both the early and the delayed phase after HO preconditioning. NF-κB may be a common factor in mediating injury-reperfusion tolerance in both ischemic and HO preconditioning. On the other hand, several studies have demonstrated that specific inhibitors of NF-κB activation and nuclear translocation block the cardioprotective effect of IPC, suggesting that NF-κB plays a critical role in injury-reperfusion tolerance in the heart[89,90]. The cellular mechanism by which IPC activates NF-κB has been known to involve the formation of ROS, and activation of PKC and tyrosine kinase–dependent pathways[91].

Although TNF-α contributes to mediating ischemic brain injury, its expression and neuroprotective role in models of IPC have also been described. However, the role of TACE in IPC and IT is known. Madrigal et al.[92] have demonstrated that TACE inhibition blocks stress-stimulated translocation of NF-κB to the nucleus (Fig. 2-B). Thus, glutamate receptor activation may induce TACE up-regulation and subsequent increases in serum TNF-α levels, accounting for stress-induced iNOS expression via NF-κB activation (Fig. 2-C). TNF-α is released in its soluble form by TACE. TNF-α exerts some of its effects through the activation of a proinflammatory transcription factor, NF-κB[93]. Divergent signaling pathways downstream of TNFR1 (p55), which must ubiquitously express TNF receptor, lead to either survival or cell death (Fig. 2-B, A2)[94].

TNF-α may induce apoptosis via the TNFR1 death domain and adaptor proteins that lead to caspase activation, whereas it can increase cell survival via activation of NF-κB–dependent genes (Fig. 2-A2)[95]. Indeed, there is much evidence demonstrating a TNF-α–induced neuroprotective and antiapoptotic action on neural cells[31]. Both activation of TACE enzymatic activity and increased TACE expression play an important role in this antiapoptotic effect (Fig. 2-B). The neuroprotective effect of TNF-α is mediated via activation of NF-κB, as the proteosome inhibitor MG132 is able to revert it. Interestingly, MG132 also induced apoptosis in control cells, suggesting that this pathway is necessary for cell survival[86].

Strong evidence points to NF-κB as a mediator of the protective actions of TNF-α[31]. Cardenas et al.[96] demonstrated that the neuroprotection effect of TACE-induced TNF-α release in a setting of mild neuronal damage using cortical cultures exposed to glutamate, which may correlate with IPC, is mediated via activation of NF-κB[86]. Other studies have described NF-κB activation in TNF-α induction of IT[97]. NF-κB would increase the expression of antiapoptotic proteins such as Bcl2 or decrease the activation of proapoptotic caspase-3 since the presence of both processes has been demonstrated after IPC (Fig. 2-B)[98].

Signaling cascades involved in cell survival are complex and although widely studied, the mechanisms behind their activation and modulation by factors including ROS are not well understood, whereas the activation of JUK and P38 by ROS has generally been implicated in promoting cell death[99]. The PI(3)K/Akt/NF-κB pathway is also a key player in preventing apoptosis and induces activation of Akt, and its downstream effectors have been shown to be necessary for the survival of a number of cell types, including neurons (Fig. 2-A2)[100].

TNF-α stimulates the transcription factor NF-κB in primary cortical neurons through the IKK (IkB kinase) complex[101]. Although a key role of IKK in NF-κB signaling has been established, NF-κB may be activated independent of IKK, such as glutamate and hypoxia[102]. Whether the effect is pro- or antiapoptotic depends on the stimulus, the type of cell, the activated stimulus, and the duration of NF-κB activation[103]. The PI(3)K pathway has been implicated as being involved in the activation of NF-κB through a direct interaction either between PI(3)K or Akt and IKK, or between Akt and IkB-α[104]. Wada et al. described the alterations of free radical oxygen (FRO) and Bcl2, an inhibitor of apoptosis, after repeated HBO exposure in gerbils, which correlated with increased neural survival (Fig. 2-A2)[22]. Finally, recent findings suggest other possible mechanisms: (1) up-regulation of Bcl2 via NF-κB attenuates the generation of ROS[105]; (2) the colocalization of Bcl2 with calcium channels on mitochondria, ER, and nuclear membrane promote calcium homeostasis[106]; (3) Bcl2 plays a critical role in the control of apoptosis and necrosis (Fig. 2-B, A2)[107].

## HO AND ANTIOXIDANT ENZYMES

Strong studies have suggested that oxidant stress resulting from the generation of ROS is present in the brain after ischemia, HO, and hypoxia[108]. ROS are generated as a by-product of both normal and aberrant metabolic processes that have molecular oxygen and NO substrates (Fig. 2-C). Cellular defense systems against ROS include enzymes that convert ROS to less reactive species (catalase, superoxide dismutase, glutathione [GSH] peroxidase) and antioxidants that quench the ROS[109]. On the other hand, normobaric HO can produce FRO and increase antioxidant enzyme activity in the brain. FRO induced by normobaric HO explains the underlying possible mechanism by which normobaric HO induces antioxidant enzyme activities. Normally, antioxidant enzymes maintain a high ratio of GSH/GSSH for antioxidant defense in the brain tissue. NF-κB is activated by various intracellular signals, including cytokines, TNF-α, neurotrophic factors, and neurotransmitters (Fig. 2-B). Inhibition of NF-κB prior to HO abolishes protection[110]. NF-κB is a transcription factor for TNF-α which, in turn, induces activation of NF-κB in a positive feedback loop[111].

## HO AND SUPEROXIDE DISMUTASE

There are three major endogenous superoxide dismutases. Cu,Zn-superoxide dismutase is principally found in the cytosolic and lysosomal fractions, and mitochondrial intermembrane space[112]. Mn-superoxide dismutase is found in the mitochondrial matrix. Both Cu,Zn-superoxide dismutase and Mn-superoxide dismutase are abundant in the neural tissue and, for this reason, have received the greatest scrutiny. Extracellular superoxide dismutase is also expressed in the brain, but in substantially lower concentrations than Cu,Zn-superoxide dismutase and Mn-superoxide dismutase[113]. Cu,Zn-superoxide dismutase overexpression reduces ischemic damage from ischemia-reperfusion[114]. Cu,Zn-superoxide dismutase overexpression has been shown to inhibit postischemic MAPK[115], the bad cell death signaling pathway[116], caspase activation[117], and fragmentation[118]. These data indicate a potential proapoptotic role for superoxide in ischemia-reperfusion (Fig. 2-B, A2). Superoxide anions can be abated by superoxide dismutase overactivity and overexpression, by pretreatment with HO or ROS. Therefore, ischemia-reperfusion presents numerous opportunities for formation of reactive oxygen/nitrogen species and resultant tissue injury. It has been shown that overactivation of superoxide dismutase induced by intermittent and prolonged normobaric HO partly reduces ischemic-reperfusion injury via dismutation of superoxide derived from various sources at different stages of reperfusion[5]. On the other hand, the increase of FRO caused by HBO and superoxide dismutase was associated with decreased expression of hypoxia inducible factor-1–α (HIF-1α), leading to improved BBB function via decreased endothelial vascular growth factor (Fig. 2-A2)[119].

## HO, CATALASE, AND GLUTATHIONE PEROXIDASE

Superoxide dismutase dismutates superoxide to $H_2O_2$ and oxygen. $H_2O_2$ has modest oxidative potential and can freely cross cell membranes. $H_2O_2$ can be converted to hydroxyl radicals[5,120]. Therefore, elimination of $H_2O_2$ is critical for the efficacy of superoxide dismutase in reducing oxidative stress. Catalase and glutathione peroxidase serve this purpose. These enzymes are present in the brain, although glutathione peroxidase activity is sevenfold greater than that of catalase[121]. Further, while glutathione peroxidase is present in the cytosol, catalase is located mainly in peroxisomes. Both glutathione peroxidase overexpression and knock-out mice have been studied in the context of focal cerebral ischemia-reperfusion. Overactivation of glutathione peroxidase and catalase reduce necrotic and apoptotic cell death, astrocytic/microglial activation, and inflammatory cell infiltration[120] (Fig. 2-B, A2). The progeny of cross-breeding a glutathione peroxidase knock-out and a Cu,Zn-superoxide dismutase overexpressor caused a loss of protection that was otherwise provided by overexpression of Cu,Zn-superoxide dismutase. However, the glutathione peroxidase knock-out alone was insufficient to worsen cerebral ischemia-reperfusion injury (Fig. 2-B, A2)[120].

Overactivation of glutathione peroxidase and catalase induced by intermittent and prolonged normobaric HO partly reduce ischemia-reperfusion injury[5].

## GLUTATHIONE REDUCTASE

Glutathione is a tripeptide (γ-L-glutamyl-L-cysteinylglycine) that is the reductant for glutathione peroxidase. Oxidation of the cysteine sulfhydryl groups joins two GSH molecules with a disulfide bridge to form glutathione disulfide (GSSG). Glutathione reductase catalyzes recovery of glutathione[120].

Normally, the brain maintains a high ratio of GSH/GSSG for antioxidant defense (Fig. 2-B). Depletion of total glutathione and a decreased GSH/GSSG ratio are markers for oxidative stress in the ischemic brain and as long as 72 h may be required to restore concentrations to normal values following an ischemic insult[122]. Also, it has been shown that HBO preconditioning decreased mortality rate, improved neurological recovery, lessened neuronal injury, reduced the level of MDA, and increased the antioxidant activity of catalase and superoxide dismutase. The up-regulation of antioxidant enzyme activity by HBO preconditioning plays an important role in the generation of tolerance against brain ischemic-reperfusion injury (Fig. 2-B, A2)[123].

## HO AND INFLAMMATION

Polymorphonuclear neutrophils (PMN) can contribute to secondary brain injury by reducing microvascular blood flow, initiating thrombosis, and by releasing oxygen free radicals[124]. Activation of PMN by surface molecules on the endothelial cells adjacent to ischemic neurons and supporting cells triggers infiltration of these inflammatory cells into the area of infarction and penumbra. It has been shown that one of the other possible protective mechanisms of HO and HBO is the reduction of inflammatory-mediated secondary brain injury resulting from the activation and infiltration of PMN[125]. Namely, inhibition of β-2 integrin and down-regulation of ICAM-1 expression caused by HBO may reduce PMN infiltration in brain injury area (Fig. 2-A1)[126,127].

In the focal ischemic model, glucose and pyruvate concentration are increased after reperfusion. All metabolites decreased gradually to control levels at 24 h after reperfusion. HO decreases glucose, pyruvate, and glutamate during reperfusion[128]. Therefore, HO has useful effects on infarction and dynamic energy metabolic disturbance at the phase of reperfusion (Fig. 2-A1).

In newborn rats, HO can promote the proliferation of neural stem cells in the subventricular zone within 24 h after ischemia[129]. Proliferation and differentiation are regulated by external factors, such as cytokines, neurotransmitters, hormones, and autogenes[20,130]. On the other hand, Freiberger et al.[131]

have shown that a comparison of HBO vs. hypoxia cerebral preconditioning in neonatal rats results in neuroprotection. They demonstrated that oxidative stress is expressed differently in the two, implying distinct preconditioning mechanisms. Although the mechanism of HO preconditioning requires further elucidation, oxidants generated during the brief period of highly elevated $PO_2$ are important for triggering many signaling systems that are more oxidant sensitive, including various kinases, e.g., p38MAPK, and transcription factor, e.g., HIF-1 (Fig. 2-B, A2). Although current data suggest that HO-mediated neuroprotection is partly due to the induction of glutamate transporter expression, other mechanisms may be participating. HO and HBO can produce angiogenesis and increase vessel density in the brain[132], and can inhibit ICAM-1 expression and neutrophil accumulation[133,134]. In addition, HBO inhibits matrix metalloproteinase-9 and occludin degradation in cerebral microvessels in experimental stroke[135,136].

In conclusion and future work, many questions remain unsolved in HO preconditioning. For example, does our chronic HO preconditioning increase the susceptibility to stroke or related excitotoxicity diseases? Does it lead to changes in glutamate release? Does it modify the interactions between glutamate receptors? Does it change affinity, activity, and expression of the mGlutR, NCX, and $Ca^{2+}$ ATPase? And most important, can we use or mimic HO preconditioning and prevent the awful tragedies that stroke, aneurysm surgery, and acute ischemia can cause?

## ACKNOWLEDGMENT

This study was supported by a grant from Iran National Science Foundation (INSF). We thank Professor Abolhassan Ahmadiani, Professor Ali Khoshbaten, Professor Sohrab Hajizadeh, and Dr. Bahram Rasoulian for critical review of the manuscript.
